# Effects of L-citrulline supplementation on blood pressure: A systematic review and meta-analysis

**Published:** 2019

**Authors:** Bahareh Barkhidarian, Masoud Khorshidi, Sakineh Shab-Bidar, Baran Hashemi

**Affiliations:** 1 *Department of Nutrition, Mashhad University of Medical Sciences, Mashhad, Iran*; 2 *Department of Clinical Nutrition, School of Nutritional Sciences and Dietetics, Tehran University of Medical Sciences (TUMS), Tehran, Iran*; 3 *Department of Community Nutrition, School of Nutritional Sciences and Dietetics, Tehran University of Medical Sciences (TUMS), Tehran, Iran*; 4 *Rajaie Cardiovascular Medical and Research Center, Iran University of Medical Sciences, Tehran, Iran*

**Keywords:** L-citrulline, Blood pressure, Supplementation

## Abstract

**Objective:**

We aimed to conduct a systematic review and meta-analysis of clinical trials that examined the effects of L-citrulline supplementation on blood pressure (BP).

**Materials and Methods:**

We searched MEDLINE, SCOPUS, PUBMED and Google scholar databases from inception to November 16, 2017 and 811 papers were identified, of which 8 trials with 10 data sets met the inclusion criteria. Inclusion criteria were: (1) application of randomized clinical trial with either crossover or parallel designs; (2) studies conducted in adults (≥18 y); (3) oral supplementation with L-citrulline compared to control group; (4) expression of sufficient data about systolic and diastolic BP at baseline and at the end of the study in each group. BP effects were pooled by random-effects models, with trials weighted by inverse variance.

**Results:**

The included studies’ sample size ranged between 12 and 34 subjects. The mean age of the participants in these trials ranged between 22 and 71 years. Dosage of L-citrulline supplementation varied from 3 to 9 g/day. Duration of the intervention ranged between 1 and 17 weeks. The pooled changes in systolic and diastolic BP were (MD, −4.10 mm Hg; 95% CI [−7.94, -0.26]; p=0.037) and (MD −2.08 mm Hg; 95% CI [−4.32, 0.16]; P=0.069), respectively. The subgroup analysis showed a significant diastolic BP reduction in studies that used doses of ≥6 g/day (MD −2.75 mm Hg; 95% CI [−5.37, -0.12]; p=0.04).

**Conclusion:**

Our results suggest that L-citrulline supplementation may reduce systolic BP. A significant reduction in diastolic BP was observed only in the studies that used doses ≥ 6 g/day.

## Introduction

According to the World Health Organization (WHO) report, hypertension globally affects approximately 40% of adults aged 25 and above (World Health Organization, 2013). Hypertension is one of the most common diseases that lead to office visits or hospitalization, and is the major cause of stroke, congestive heart failure (CHF), myocardial infarction (MI), peripheral vascular disease, and overall mortality. Hypertension is responsible for at least 45% of deaths due to heart diseases and 51% of stroke deaths (World Health Organization, 2008).

L-Citrulline (L-cit) (C_6_H_13_N_3_O_3_) is a non-protein amino acid that has been investigated for lowering the blood pressure. L-cit is derived from *Citrullus* *vulgaris* that is watermelon, and L-cit was first isolated from this plant in the 1930s (Curis et al., 2005 ▶). Until recently, it was considered only an intermediate chemical in the urea cycle. However, recent studies have indicated the importance of this amino acid in cellular metabolism and organs function.

Recent studies have shown that L-cit has the potential to increase the plasma nitrite and NO level. (Schwedhelm et al., 2008 ▶; Morita et al., 2014 ▶). Many types of cells which are able to metabolize arginine into NO, are able to uptake L-cit. This explains why L-cit causes effects such as reducing the tonicity of the muscle in blood vessels similar to NO (Raghavan and Dikshit, 2001 ▶). Another study showed that the effects of L-cit on retinal arterioles dilation in rats is mediated via both NO and prostaglandin-dependent pathways (Mori et al., 2015 ▶).

Several studies have investigated the effect of L-cit supplementation on the blood pressure. Despite non-significant changes in the blood pressure in some studies (Figueroa et al., 2016 ▶; Balderas-Munoz et al., 2012 ▶; Ochiai et al., 2012 ▶), other experiments have found the beneficial impact of L-cit supplementation on either SBP (systolic blood pressure) or DBP (diastolic blood pressure (Wong et al., 2015 ▶; Wong et al., 2016 ▶; Gonzales et al., 2017 ▶; Sanchez-Gonzalez et al., 2012 ▶; Orozco-Gutiérrez et al., 2010 ▶). Figueroa and coworkers showed that L-cit attenuated the SBP in response to the cold pressor test (Figueroa et al., 2010 ▶). In a recent study done by Figueroa and colleagues, it was stated that 2-week supplementation with L-cit protected overweight and obese men from cardiac afterload elevation when confronting physical stress (Figueroa et al., 2016 ▶). In obese pre-hypertensive and hypertensive subjects, L-cit supplementation reduced both blood pressure and cardiac augmentation index (Figueroa et al., 2012 ▶). Moreover, through reducing arterial stiffness, L-cit could improve leg muscle function in pre-hypertensive and hypertensive patients and attenuated cardiovascular risk (Figueroa et al., 2015 ▶). 

Also, it was shown that reduced nephron number and renal dysfunction in rat’s adult offspring due to maternal calorie restriction, may be reversed by L-cit and maternal L-cit supplementation exacerbates the elevation of blood pressure (Tain et al., 2010 ▶). Due to controversies regarding the effect of L-cit on the blood pressure, a meta-analysis of randomized clinical trials was conducted to determine the direction and magnitude of the effect of L-cit on the blood pressure. 

## Materials and Methods

**Search strategy and study selection**

The present meta-analysis followed the Preferred Reporting Item for Systematic Reviews and Meta-analysis (PRISMA) guidelines in writing this meta-analysis (Moher et al., 2009 ▶).

Literature search was conducted in MEDLINE, SCOPUS, PUBMED and Google scholar databases from inception to November 16, 2017. The following search terms were used: L-citrulline (MeSH term) OR L-citrulline (title/abstract) OR L-cit (title/abstract) OR citrulline malate (title/abstract) AND aortic blood pressure (MeSH term) OR aortic blood pressure (title/abstract) OR blood pressure (MeSH term) OR blood pressure (title/abstract) OR diastolic blood pressure (title/abstract) OR systolic blood pressure (title/abstract) OR mean arterial pressure (title/abstract) OR hypertension (title/abstract) in PubMed and ‘L-citrulline’ (Article title, Abstract, Keywords) and ‘blood pressure’ (Article title, Abstract, Keywords) in Scopus, with no language or publication date restriction. This search was also followed by hand search of the reference list of RCTs to include other potentially eligible trials. 

**Eligibility and selection criteria**

The title and abstract of studies were separately reviewed by two researchers to choose relevant studies, and discrepancies were resolved through discussion with the third researcher. Human studies were eligible for inclusion if they fulfilled the following criteria: (1) Application of randomized clinical trial with either crossover or parallel designs; (2) Studies conducted in adults (age ≥18 years old); (3) Oral supplementation with L-cit compared to control group; (4) Presentation of enough data about intended variables (SBP and DBP) at baseline and at the end of study in each group. 

*The following studies were excluded*

(1) studies with designs other than randomized clinical trial; (2) combined uses of L-cit and other substances; (3) using a mixture of L-cit with other substances and foods rich in citrulline including watermelon; (4) studies not relevant to research question; (5) studies with inadequate statistical information or those that did not meet the inclusion criteria.

**Data extraction**

After searching the databases, two reviewers separately screened included studies. The following characteristics were extracted from each included publication: study first author, year of publication, the country where the study was conducted in, study design (parallel or cross over), characteristics of study population (e.g. age, sex and health condition), number of participants, dose of administrated L-cit, intervention duration and outcome data. Moreover, mean and SD of the SBP and DBP at baseline and at the end of intervention were extracted. In case of cross-over trials, only data from the first part of the study (before washout period) was used for analysis.

**Quality assessment**

The quality of publications was assessed by Jadad scales that assign scores for randomized controlled trial (Jadad et al., 1996). Studies with scores ≥3 were classified as "high" quality studies and those of ≤2 were classified as "low" quality studies (Table 2).

**Data synthesis and statistical analysis**

All analyses were conducted using Stata software, version 14 (Stata Corp, College Station, Texas, USA). The changes in mean and standard deviation (SD) of the SBP and DBP between baseline and post-intervention, for the intervention and control groups, were considered for analysis. SD was calculated when the information was reported as standard error of means (SEM) by multiplying SEM by square root of the sample size: SD=SEM × √n. SD of the changes in blood pressure from baseline, if not reported, was calculated using the following formula (Wu et al., 2017 ▶).

SD_ changes_= √ SD _baseline_^2^ + SD _final _^2 ^– (2× 0/8 × SD _baseline _× SD _final_) 

To calculate the overall effect size, weighted mean difference (WMD) and 95% confidence interval (CI) were calculated. 

Heterogeneity was determined using Cochrane’s Q test (Higgins et al., 2003 ▶). Since we found heterogeneity (I²≥50%), we used a random-effects model. In order to identify potential sources of heterogeneity, subgroup analyses were carried out; these subgroups included studies design, trial duration, mean age of participants and dose of L-cit supplementation. 

Sensitivity analysis was performed to explore the extent to which inferences might depend on a particular trial using the leave-one-out method. 

Publication bias was tested by visual inspection of funnel plots. Statistical assessment of the asymmetry in funnel plot was performed using Egger’s regression asymmetry test and Begg’s adjusted rank correlation test. For all analysis p-values <0.05 was considered statistically significant. 

## Results

**Literature search and study characteristics**

**Figure 1:**
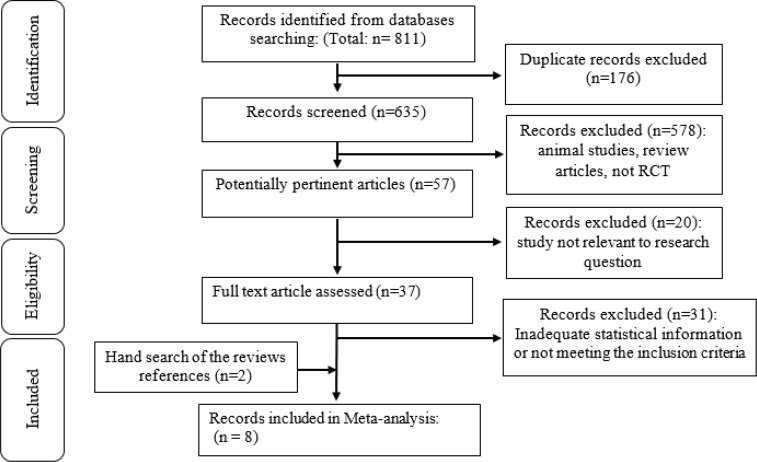
Flow chart of study selection

The flowchart of literature search is shown in Figure 1. Out of a total of 811 publications, after removing duplicated one, 635 records remained for title and abstract screening. Of those studies, 578 records were excluded because they were animal studies or review articles and not RCT’s. Of 57 remaining studies, 20 were not relevant to our meta-analysis question. Thirty seven full-text articles were evaluated in details. Finally, 8 studies with 10 data sets investigating the effect of L-cit supplementation on blood pressure were included (Figueroa et al., 2010 ▶; Figueroa et al., 2016 ▶; Ochiai et al., 2012 ▶; Wong et al., 2015 ▶; Wong et al., 2016 ▶; Sanchez-Gonzalez et al., 2012 ▶; Gonzales et al., 2017 ▶; Balderas-Munoz et al., 2012 ▶).

Among these trials, the smallest population size was comprised of 12 participants while the largest population size included 34 participants. Included trials were published between 2009 and 2017, and were conducted in the USA (Figueroa et al., 2010 ▶; Gonzales et al., 2017 ▶; Wong et al., 2015 ▶; Wong et al., 2016 ▶), Israel (Figueroa et al., 2016 ▶), Japan (Ochiai et al., 2012 ▶) and Mexico (Balderas-Munoz et al., 2012 ▶). The mean age of participants in these trials ranged between 22 and 71 years. Dosage of L-cit supplementation varied from 3 to 9 g/day. Duration of intervention ranged between 1 and 17 weeks. Four trials were crossover (Gonzales et al., 2017 ▶; Figueroa et al., 2016 ▶; Sanchez-Gonzalez et al., 2012 ▶; Figueroa et al., 2010 ▶) and the other four trials were parallel trials (Ochiai et al., 2012 ▶; Wong et al., 2015 ▶; Wong et al., 2016 ▶; Balderas-Munoz et al., 2012 ▶). The methodological quality score of selected papers ranged from 2 to 4 as assessed by the Jadad scale (Table 2). Other characteristics of included studies are shown in Table 1.

**Table 1:** Characteristics of studies included in this meta-analysis

**First Author and year**	**Country**	**Study population**	**sex**	**dose (g/day)**	**Duration (week)**	**Sample size**	**Mean age**	**Study design**	**Jadad score**	**Results**
	Japan	healthy subjects	M	5.6	1	15	58	p	3	SBP and DBP did not change significantly
	USA	healthy subjects	F	6	8	23	58	p	3	SBP and DBP decreased significantly
**Figueroa et al. 2009**	USA	healthy subjects	M	6	4	17	22	c	3	SBP and DBP did not change at rest after intervention
**Figueroa et al. 2009**	USA	healthy subjects	M	6	4	17	22	c	3	SBP decreased significantly but DBP not changed significantly during CPT after intervention
Wong et al. 2015 ▶*	USA	healthy subjects	F	6	8	27/41	58	p	3	SBP and DBP decreased significantly
	USA	healthy subjects	F	6	2	13	70	c	4	SBP and DBP did not change significantly.
	USA	healthy subjects	M	6	2	12	71	c	4	SBP did not change significantly. DBP decreased significantly
	Israel	healthy subjects	M	6	2	16	24	c	4	SBP and DBP did not change significantly
	USA	healthy subjects	M	9	2	16	23	c	2	SBP and DBP decreased significantly
**Munoz et al. 2012**	Mexico	Heart Failure	M/F	3	17	34	67	p	2	SBP and DBP did not change significantly

SBP, systolic blood pressure; DBP, diastolic blood pressure, M: male, F: female, P: Parallel, C: crossover

* Data of the two groups from three groups of the study by Wong et.al. 2015 were used for analysis. The total number of patients were 4 but 27 were analyzed.

**Table 2:** Jadad scale of the included studies

**Reference**	**Randomization is mentioned**	**Appropriateness of Randomization**	**Blinding is mentioned**	**Appropriateness of blinding**	**An account of all patients or description of withdrawal or drop out**	**Total**
	1	0	1	0	1	3
	1	1	0	0	1	3
**Figueroa et al. 2009**	1	0	1	0	1	3
	1	1	0	0	1	3
	1	0	1	1	1	4
	1	0	1	1	1	4
	1	0	1	0	0	2
**Munoz et al. 2012**	1	0	0	0	1	2

**L-cit and SBP**

**Figure 2:**
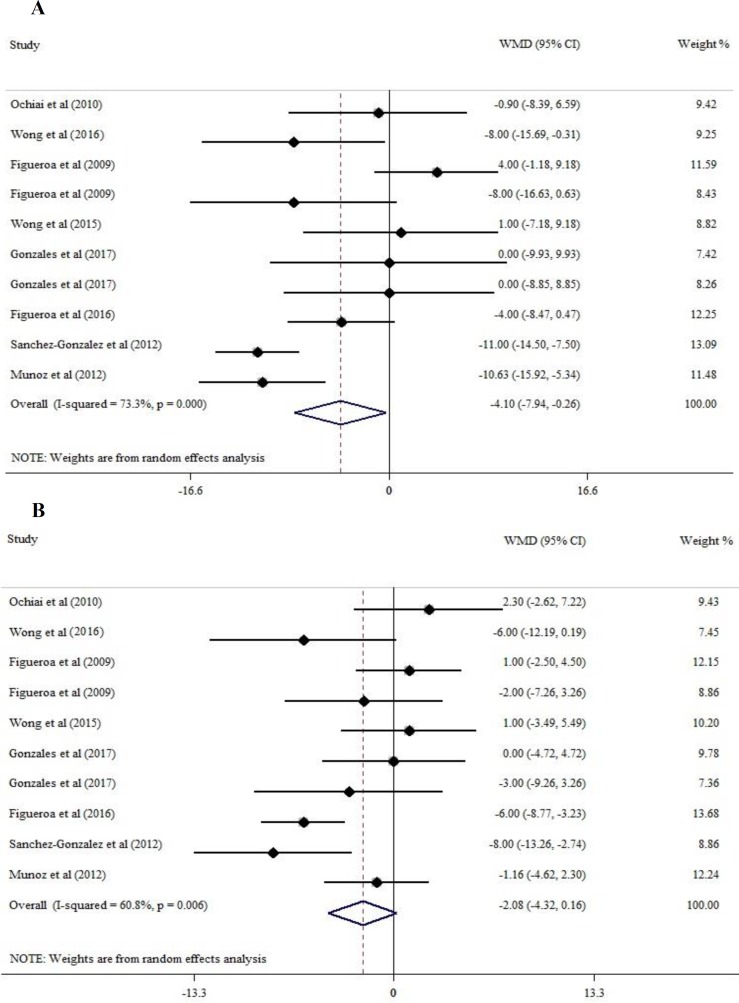
Forest plot of WMD of SBP (A) and DBP (B) at baseline and after L-citrulline supplementation and their 95% confidence intervals

As there was significant heterogeneity among studies (I^2^=73.3%, p=0.00), we used a random-effects model to estimate the pooled MD. Using this model, the pooled effect size of L-cit supplementation on SBP versus control was calculated (MD, −4.10 mm Hg; 95% CI [−7.94, -0.26]; p=0.037). The result showed that supplementation with L-cit has significant effects on lowering SBP (Figure 2). 

Sub-group analysis indicated that the duration of study (less or more than 4 weeks), dose of L-cit <6 g/day or ≥6 g/day and mean age of participants (20-25 years or >55 years) were not the potential sources of heterogeneity (Table 3). 

**Table 3:** Results of subgroup analysis of randomized controlled trials included in this meta-analysis of the effect of L-citrulline supplementation on blood pressure

**Variable **	**Mean age**		**Dose of supplementation**		**Trial duration**	
** SBP **	**> 55 years**	**20-25 years **	**≥ 6 g/day **	**< 6 g/day **	**≥ 4 weeks**	**< 4 weeks **
**No. of comparison**	4	6	2	8	5	5
**WMD (95% CI)**	-4.709 (-7.940, 2.243)	-3.761 (-8.273, 0.752)	-6.141 (-15.647, 3.366)	-3.505 8.013, 1.003))	-4.125 (-9.145, 0.895)	-4.240 (-10.723, 2.244)
**p value**	0.184	0.102	0.206	0.127	0.107	0.200
**I** ^2^ ** (%)**	87	51.6	76.9	75.3	70.2	78.3
**p-heterogeneity**	>0.001	0.066	0.038	> 0.001	0.009	0.001
**DBP**						
**No. of comparison**	4	6	2	8	5	5
**WMD (95% CI)**	-3.664 (-4.315 , 0.164)	-0.702 (-2.728, 1.325)	0.111 (-3.158, 3.380)	-2.750 (-5.372, -0.128)	-3.057 (-6.802, 0.688)	-0.765 (-2.824 , 1.294)
**p value**	0.080	0.497	0.947	0.040	0.11	0.466
**I** ^2^ ** (%)**	5.8	76.1	21.3	62.4		
**p-heterogeneity**	0.362	0.006	0.260	0.010		

CI, conﬁdence interval; WMD, weighted mean differences, SBP, systolic blood pressure, DBP, diastolic blood pressure

**Figure 3:**
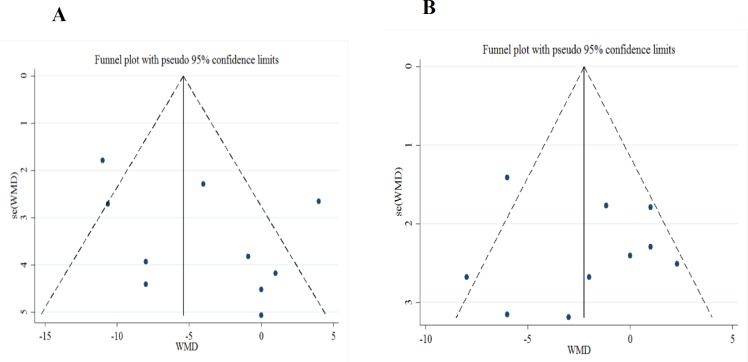
Funnel plot for SBP (A) and DBP (B).

Sensitivity analysis examining the effect of an individual trial on the combined effect size yielded a range from -3.08 (95%CI=−6.71, 0.54) to -5.42 (95% CI=−8.78, -2.07) for SBP. No individual study had appreciable impacts on the overall combined effects (Table 4).

**L-cit and DBP**

In meta-analysis of 8 trial (10 data sets) supplementation with L-cit decreased DBP but this decrement was not significant (MD −2.08 mm Hg; 95% CI [−4.32, 0.16]; p=0.069); however, this analysis was associated with significant heterogeneity (I^2^=60.8%, p=0.006) (Figure 2). 

When a subgroup analysis was done for dosage (<6 g/day or≥6 g/day), results for DBP showed greater efﬁcacy of L-cit supplementation at the higher dose (MD -2.750 mm Hg; 95% CI [-5.37, -0.128]; p=0.04) (Table 3). The results of sub-group analysis indicated that none of pre-defined criteria were potential sources of heterogeneity (Table 3). 

As Table 4 shows, sensitivity analysis indicated that none of the trial has appreciable impact on overall combined effect size, the result of sensitivity analysis for DBP is in range of -1.35 (95%CI=−3.41, 0.71) to -2.52 (95% CI=−4.80, -0.24). 

**Publication bias**

 Based on visual inspection of the funnel plot (Figure 3), neither changes in SBP nor changes in DBP revealed potential publication bias. No evidence of publication bias was detected by the Begg’s test (z=0.89, 2-tailed p=0.31, for SBP), (z=0.54, 2-tailed p=0.592, for DBP). Similarly, Egger’s linear regression test did not show any evidence of publication bias (t=1.48, 2-tailed p=0.177 for SBP) and (t=0.34, 2-tailed p=0.74 for DBP).

**Table 4:** Sensitivity analyses of the impact of each trial on pooled effect size

**Study omitted**	**WMD (95 % CI)**
Ochiai et al (2010)	-4.41 (-8.54, -0.28)
Wong et al (2012)	-3.66 (-7.85, 0.51)
Figueroa et al (2009)	-5.42 (-8.78, -2.07)
Figueroa et al (2009)	-3.71 (-7.85, 0.43)
Wong et al (2015)	-4.58 (-8.62, -0.55)
Gonzales et al (2017)	-4.41 (-8.46 , -0.36)
Gonzales et al (2017)	-4.45 (-8.52 , -0.38)
Figueroa et al (2016)	-4.045 (-8.51, 0.42)
Sanchez-Gonzalez et al (2012)	-3.08 (-6.71, 0.54)
Munoz et al (2012)	-3.24 (-7.36, 0.86)
Ochiai et al (2010)	-2.52 (-4.80, -0.24)
Wong et al (2012)	-1.75 (-4.10, 0.58)
Figueroa et al (2009)	-2.50 (-4.85, -0.14)
Figueroa et al (2009)	-2.08 (-4.54, 0.37)
Wong et al (2015)	-2.42 (-4.80, -.045)
Gonzales et al (2017)	-2.30 (-4.74, 0.13)
Gonzales et al (2017)	-2.00 (-4.42, 0.41)
Figueroa et al (2016)	-1.35 (-3.41, 0.71)
Sanchez-Gonzalez et al (2012)	-1.50 (-3.69, 0.67)
Munoz et al (2012)	-2.20 (-4.76, 0.35)

**Meta- regression analysis**

Meta-regression analysis was performed to evaluate the association between changes in SBP and DBP and potential moderator variables including the administered dose and duration of the study. The results suggested that the dose of L-cit (p=0.08 and p=0.2 for SBP and DBP respectively) and intervention duration (p=0.3 for SBP, p=0.72 for DBP) had no effects on pooled estimate (Table 5). 

**Table 5:** Characteristics associated with net change in blood pressure (BP): univariate meta-regression analysis

	**95% CI**	**p**	**95% CI**	**p**
**Dose **	-3.28 to 2.76	p=0.84	-2.91 to 0.74	p=0.20
**Duration **	-1.28 to 0 .46	P=0.30	-0.46 to 0.63	p=0.72

SBP, systolic blood pressure; DBP, diastolic blood pressure; and CI, confidence interval.

## Discussion

Although several clinical trials have assessed the impact of L–cit on hemodynamic parameters, controversial results have been achieved in terms of the effects of L-cit on the blood pressure because significant changes have not been reported consistently. To the best of our knowledge, this is the first meta-analysis that examined the effect of L-cit supplementation on the blood pressure. 

In our study, pooled analysis showed that L-cit supplementation causes a modest but significant reduction of about 4 mmHg in SBP compared to the placebo group. Although this effect seems small, it is in the opposite direction of the increasing prevalence of high blood pressure. Moreover, our subgroup analysis showed that DBP reduction was dose-dependent. A dose of ≥6 g/day reduced the DBP. This effect was consistent among the included studies. In one study, 6 g/day of L-cit did not diminish the DBP during the cold pressor test (Figueroa et al., 2012 ▶). In contrast, another exposure to cold indicated a significant reduction in BP after L-cit supplementation (Sanchez-Gonzalez et al., 2012 ▶). This might be attributed to different durations of cold exposure (2 versus 30 min). The heart rate response to cold water immersion was shown to reduce in later compared to earlier immersion (Tipton, 1989 ▶). Furthermore, it may be explained by the time latency between the administration of the last dose and BP measurement. In the report published by Moinard and coworkers, L-arginine returned to its basal level 5-8 hr after acute L-cit supplementation in old men (Moinard et al., 2016 ▶). In the study in which L-cit supplementation did not reduce the DBP, participants took L-cit twice a day; hence, the last dose was taken 9-12 hr before morning measurements. In the study done by Gonzales and coworkers on old women, L-cit supplementation did not reduce the DBP in contrast to old men (Gonzales et al., 2017 ▶); which may be explained by gender difference. As old women have lower estrogen levels, they have reduced transportation of arginine into the vasculature or lower intracellular activity. Thus, women may need higher doses of L-cit or longer duration of supplementation. Furthermore, nitric oxide (NO) donors reduce vascular smooth muscle tone in the peripheral arteries (Gamboa et al., 2016 ▶; Bailey et al., 2015 ▶; Chowdhary et al., 2002 ▶). It was shown that DBP, but not SBP, is positively related to the activity of the sympathetic nerve in the resting muscle in the elderly men. Thus, supplementation with L-cit may reduce the sympathetic vasomotor tone by increasing smooth muscle relaxation (Hart et al., 2009 ▶). 

Among other NO donors studied for their effect on BP, L-cit is completely absorbed through the intestine and it is not affected by the hepatic metabolism (Wijnands et al., 2015 ▶; Breuillard et al., 2015 ▶). L-cit supplementation increases NO bioavailability by increasing L-arginine via L-arginine/ L-cit pathway (Moinard et al., 2016 ▶; Vanhoutte et al., 2016 ▶). There is evidence supporting that L-cit supplementation is a more efficient intervention for increasing NO bioavailability than L-arginine (Ochiai et al., 2012 ▶; Moinard et al., 2016 ▶; Wijnands et al., 2015 ▶; Breuillard et al., 2015 ▶). L-cit supplementation was found to increase the cGMP urinary excretion. CGMP is a byproduct of NO-stimulated guanylyl cyclase in the smooth muscle which results in reduced sympathetic activity and vasodilation. Acute and chronic L-cit supplementation may also increase the baroreflex sensitivity (Bailey et al., 2015 ▶). In addition, L-arginine may be the substrate of some neurons within the baroflex arc. NO is an angiotensin converting enzyme inhibitor (ACEI) (Balderas-Munoz et al., 2012 ▶). Since baroreceptors’ function is essential for the impact of adrenergic stress on the cardiovascular system to maintain adequate blood pressure levels, NO availability by L-cit supplementation might elevate the baroreceptor sensitivity. 

We observed a considerable heterogeneity across the included trials in the present meta-analysis. This significant heterogeneity might be attributed to the age of participants, study duration, and differences in administered doses of L-cit. In young subjects, there is a balance among the factors contributing to BP regulation (Hart et al., 2009 ▶); in this regard, the higher sympathetic activity was found to be associated with lower cardiac output and lesser vascular responsiveness to α-adrenergic agonists, which limited the impact of high sympathetic activity on the blood pressure, but such a balance was not observed in older subjects (Hart et al., 2009 ▶).

Moreover, the highest dose of L-cit (9 g/kg), in the studies included in this meta-analysis, had greatest effect. However, it is not known whether higher doses cause more marked BP reduction. Further studies are still needed to explore the potential dose–response effect of L-cit on the BP.

There were some limitations in our meta-analysis. Most studies that were included were performed within ≤4 weeks and had small population size and none recruited more than 34 subjects. Furthermore, the population was almost limited to healthy individuals, so, we cannot generalize our findings to high blood pressure patients. We only included resting blood pressure data in our analysis and the effect of L-cit on BP after physical activity is unknown. 

In the sensitivity analysis, we observed no changes in our results by excluding each trial; this supports the robustness of our findings. Also, based on Jadad score most of the included studies were of high quality in terms of methodology.

Large double-blind clinical trials are required to clarify the effect of L-cit intake on the blood pressure in pre-hypertensive and hypertensive patients. In addition, assessing the time course of BP changes to assess the sustained effect of supplementation is suggested. While it is still premature to recommend L-citrulline supplementation, a healthy diet including L-cit rich foods such as watermelon, may contributes to prevention of hypertension. 

In conclusion, our results suggest that L-cit supplementation may reduce the SBP. Significant reductions in DBP were observed only in the studies that used doses of ≥6 g/day.

## Conflicts of interest

The authors declared that there were no conflicts of interest.
